# *catena*-Poly[μ-aqua-2:1′κ^2^ *O*:*O*-aqua-2κ*O*-(2-fluoro­benzoato-1κ^2^ *O*,*O*′)(μ_2_-2-fluoro­benzoato-2′:1κ^2^ *O*:*O*′)bis­(μ_3_-2-fluoro­benzoato)-2′:1:2κ^4^ *O*:*O*,*O*′:*O*′;1:2:1′κ^5^ *F*,*O*:*O*,*O*′:*O*′-dilead(II)]

**DOI:** 10.1107/S1600536808022678

**Published:** 2008-07-23

**Authors:** Bi-Song Zhang

**Affiliations:** a College of Materials Science and Chemical Engineering, Jinhua College of Professions and Technology, Jinhua, Zhejiang 321017, People’s Republic of China

## Abstract

In the title compound, [Pb_2_(C_7_H_4_FO_2_)_4_(H_2_O)_2_]_*n*_, one Pb^II^ atom is coordinated by seven O atoms and one F atom from five 2-fluoro­benzoate ligands, and the other Pb^II^ atom is coordinated by five O atoms from four 2-fluoro­benzoate ligands and three water mol­ecules, resulting in distorted PbO_7_F and PbO_8_ polyhedra. The 2-fluoro­benzoate ligands bridge Pb atoms, giving rise to a one-dimensional chain structure extending along the [100] direction. The polymeric chains are connected *via* C—H⋯O hydrogen bonds and π–π inter­actions, with an inter­planar distance of 3.46 (1) Å. An intramolecular O—H⋯F interaction is also present.

## Related literature

For related literature, see: Morsali & Mahjoub (2005 ▶); Xiao & Morsali (2007 ▶); Zhang (2004 ▶, 2005 ▶, 2006*a* ▶,*b* ▶,*c* ▶); Zhang *et al.* (2005 ▶); Zhu *et al.* (1999 ▶).
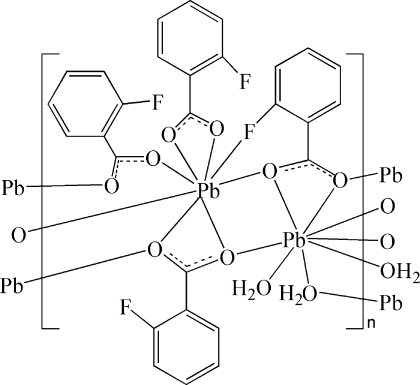


## Experimental

### 

#### Crystal data

[Pb_2_(C_7_H_4_FO_2_)_4_(H_2_O)_2_]
*M* *_r_* = 1006.84
Triclinic, 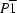

*a* = 7.1016 (14) Å
*b* = 14.794 (3) Å
*c* = 15.096 (3) Å
α = 111.56 (3)°
β = 95.32 (3)°
γ = 97.31 (3)°
*V* = 1446.2 (6) Å^3^
*Z* = 2
Mo *K*α radiation
μ = 11.71 mm^−1^
*T* = 290 (2) K
0.44 × 0.19 × 0.13 mm

#### Data collection

Rigaku R-AXIS RAPID diffractometer
Absorption correction: multi-scan (*ABSCOR*; Higashi, 1995 ▶) *T*_min_ = 0.082, *T*_max_ = 0.223
14182 measured reflections
6646 independent reflections
5329 reflections with *I* > 2σ(*I*)
*R*_int_ = 0.028

#### Refinement

*R*[*F* ^2^ > 2σ(*F* ^2^)] = 0.037
*wR*(*F* ^2^) = 0.094
*S* = 1.03
6646 reflections
397 parameters
3 restraints
H-atom parameters constrained
Δρ_max_ = 3.74 e Å^−3^
Δρ_min_ = −2.48 e Å^−3^

### 

Data collection: *PROCESS-AUTO* (Rigaku, 1998 ▶); cell refinement: *PROCESS-AUTO*; data reduction: *PROCESS-AUTO*; program(s) used to solve structure: *SHELXS97* (Sheldrick, 2008 ▶); program(s) used to refine structure: *SHELXL97* (Sheldrick, 2008 ▶); molecular graphics: *SHELXTL* (Sheldrick, 2008 ▶); software used to prepare material for publication: *SHELXL97*.

## Supplementary Material

Crystal structure: contains datablocks I, global. DOI: 10.1107/S1600536808022678/hy2143sup1.cif

Structure factors: contains datablocks I. DOI: 10.1107/S1600536808022678/hy2143Isup2.hkl

Additional supplementary materials:  crystallographic information; 3D view; checkCIF report

## Figures and Tables

**Table 1:** Selected bond lengths (Å)

Pb1—O4	2.480 (6)
Pb1—O2	2.489 (5)
Pb1—O1	2.551 (6)
Pb1—O8	2.574 (5)
Pb1—O9	2.621 (6)
Pb1—O3	2.642 (6)
Pb1—O6^i^	2.766 (6)
Pb1—F3^ii^	2.856 (8)
Pb2—O2	2.517 (5)
Pb2—O7	2.534 (6)
Pb2—O5	2.592 (6)
Pb2—O10	2.599 (6)
Pb2—O8	2.603 (5)
Pb2—O9^ii^	2.670 (6)
Pb2—O7^iii^	2.999 (6)
Pb2—O1^ii^	2.804 (5)

Symmetry codes: (i) 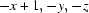; (ii) 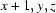; (iii) 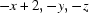.

**Table 2:** Hydrogen-bond geometry (Å, °)

*D*—H⋯*A*	*D*—H	H⋯*A*	*D*⋯*A*	*D*—H⋯*A*
O7—H7*A*⋯O6^i^	0.82	2.15	2.881 (8)	148
O7—H7*B*⋯O5^iii^	0.82	2.66	3.360 (8)	144
O10—H10*A*⋯O3^ii^	0.82	2.07	2.892 (8)	174
O10—H10*B*⋯O4	0.82	2.22	2.856 (8)	135
O10—H10*B*⋯F2	0.82	2.44	3.161 (13)	147
C19—H19⋯O3^iv^	0.93	2.56	3.364 (13)	145

Symmetry codes: (i) 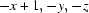; (ii) 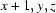; (iii) 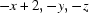; (iv) 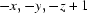.

**Fig. 1.**
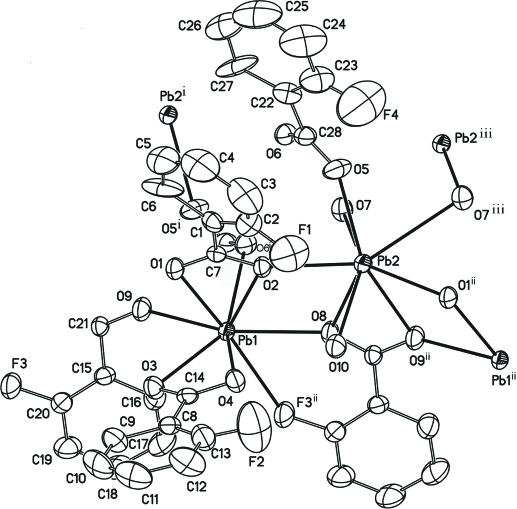
The asymmetric unit of the title compound, together with symmetry-related atoms to complete the coordination units. Displacement ellipsoids are drawn at the 35% probability level. H atoms have been omitted for clarity. [Symmetry codes: (i) 1 - x, -y, -z; (ii) 1 + x, y, z; (iii) 2 - x, -y, -z.]

**Fig. 2.**
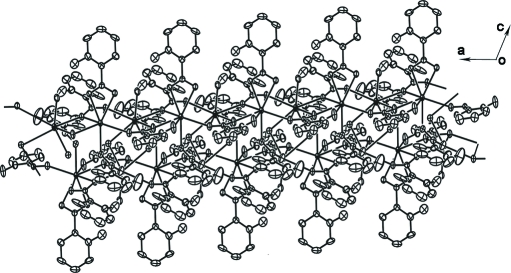
View of one-dimensional chain structure extending along the [100] direction.

**Fig. 3.**
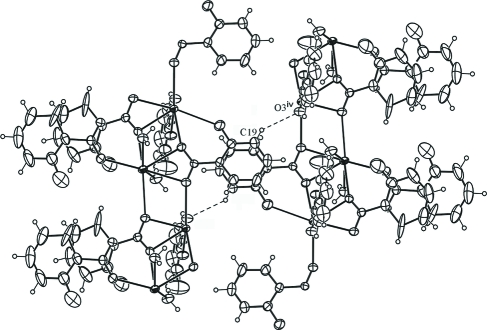
The C—H···O hydrogen bonds (dashed lines) and the π–π stacking interactions in the title compound. [Symmetry code: (iv) -x, -y, 1 - z.]

## supplementary crystallographic information

### Comment

We have studied the metal complexes of halogen-substituted benzoic acid (*X*–C_6_H_4_COOH; *X* = F, Cl, Br and I) (Zhang, 2004, 2005; Zhang *et al.*, 2005; Zhang, 2006*a*,b,c). The related crystal structures can be found, such as [Pb(phen)_n_(NO_2_)*X*] (phen = 1,10-phenanthroline; *X* = CH_3_COO^-^, NCS^-^ and ClO_4_^-^) (Morsali & Mahjoub, 2005), [Pb_3_(bpy)(H_2_O)_5_(sip)_2_].0.5bpy.2H_2_O (sip = 5-sulfoisophthalate; bpy = 2,2'-bipyridine) (Xiao & Morsali, 2007) and PbI_2_(*L*) (*L* = bpy, phen) (Zhu *et al.*, 1999). We report here the synthesis and structure of the title compound, a new one-dimensional Pb^II^ coordination polymer.

In the title compound, the Pb1 atom is coordinated by seven O atoms and one F atom from five 2-fluorobenzoate ligands to complete a significantly distorted PbO_7_F polyhedron. The Pb1—O bond lengths are in the range of 2.480 (6) to 2.766 (6) Å and the Pb1—F bond length is 2.856 (8)Å (Table 1). The Pb2 atom is coordinated by five O atoms from four 2-fluorobenzoate ligands and three water molecules to complete a significantly distorted PbO_8_ polyhedron. The Pb2—O bond lengths are in the range of 2.517 (5) to 2.999 (6)Å (Table 1). The 2-fluorobenzoate ligands bridge the Pb atoms, giving rise to a one-dimensional chain structure extending along the [100] direction (Fig. 2). There are intrachain O—H···O hydrogen bonds between the coordinated water molecules and the carboxylate O atoms of the 2-fluorobenzoate ligands (Table 2). The polymeric chains are connected *via* C—H···O hydrogen bonds and π–π stacking interactions between the benzene rings, with an interplanar distance of 3.46 (1) Å, into a two-dimensional supramolecular structure (Fig. 3).

### Experimental

Freshly prepared PbCO_3_ (0.140 g, 0.52 mmol), 2-fluorobenzoic acid (0.035 g, 0.25 mmol) in CH_3_OH/H_2_O (15 ml; 1:2 *v*/*v*) were mixed and stirred for *ca* 2 h. Subsequently, the resulting suspension was heated in a 23 ml Teflon-lined stainless steel autoclave at 423 K for 5 d. After the autoclave was cooled to room temperature, the solid was filtered off. The resulting colorless filtrate was allowed to stand at room temperature for one month, affording colorless block crystals suitable for X-ray analysis.

### Refinement

C-bound H atoms were positioned geometrically and refined as riding atoms, with C—H = 0.93Å and *U*_iso_(H) = 1.2*U*_eq_(C). H atoms of water molecules were located on a difference Fourier map and fixed with O—H = 0.82Å and *U*_iso_(H) = 1.5*U*_eq_(O). The highest residual electron density was 0.78Å from atom Pb2 and the deepest hole 0.71Å from atom Pb2.

### Crystal data

**Table:**

[Pb_2_(C_7_H_4_FO_2_)_4_(H_2_O)_2_]	*Z* = 2
*M**_r_* = 1006.84	*F*_000_ = 936
Triclinic, *P*1	*D*_x_ = 2.312 Mg m^−^^3^
Hall symbol: -P 1	Mo *K*α radiation λ = 0.71073 Å
*a* = 7.1016 (14) Å	Cell parameters from 14100 reflections
*b* = 14.794 (3) Å	θ = 3.0–27.5º
*c* = 15.096 (3) Å	µ = 11.71 mm^−^^1^
α = 111.56 (3)º	*T* = 290 (2) K
β = 95.32 (3)º	Block, colorless
γ = 97.31 (3)º	0.44 × 0.19 × 0.13 mm
*V* = 1446.2 (6) Å^3^	

### Data collection

**Table:**

Rigaku R-AXIS RAPID diffractometer	6646 independent reflections
Radiation source: rotating anode	5329 reflections with *I* > 2σ(*I*)
Monochromator: graphite	*R*_int_ = 0.028
Detector resolution: 10 pixels mm^-1^	θ_max_ = 27.5º
*T* = 290(2) K	θ_min_ = 3.0º
ω scans	*h* = −8→9
Absorption correction: multi-scan(ABSCOR; Higashi, 1995)	*k* = −19→18
*T*_min_ = 0.082, *T*_max_ = 0.223	*l* = −19→19
14182 measured reflections	

### Refinement

**Table:**

Refinement on *F*^2^	Secondary atom site location: difference Fourier map
Least-squares matrix: full	Hydrogen site location: inferred from neighbouring sites
*R*[*F*^2^ > 2σ(*F*^2^)] = 0.038	H-atom parameters constrained
*wR*(*F*^2^) = 0.094	*w* = 1/[σ^2^(*F*_o_^2^) + (0.0382*P*)^2^ + 11.3431*P*] where *P* = (*F*_o_^2^ + 2*F*_c_^2^)/3
*S* = 1.03	(Δ/σ)_max_ = 0.001
6646 reflections	Δρ_max_ = 3.74 e Å^−^^3^
397 parameters	Δρ_min_ = −2.48 e Å^−^^3^
3 restraints	Extinction correction: none
Primary atom site location: structure-invariant direct methods	

### Fractional atomic coordinates and isotropic or equivalent isotropic displacement parameters (Å^2^)

**Table:**

	*x*	*y*	*z*	*U*_iso_*/*U*_eq_	
Pb1	0.50250 (4)	0.06503 (2)	0.265288 (19)	0.02816 (8)	
Pb2	0.99101 (4)	0.11234 (2)	0.13887 (2)	0.03456 (9)	
F1	0.8986 (13)	0.3238 (6)	0.1926 (7)	0.106 (3)	
F2	0.9276 (14)	0.3801 (7)	0.5078 (9)	0.145 (4)	
F3	−0.3272 (8)	−0.0400 (6)	0.3676 (5)	0.0707 (19)	
F4	1.0244 (18)	0.2764 (11)	−0.0872 (11)	0.180 (6)	
O1	0.3833 (7)	0.1887 (4)	0.2019 (4)	0.0382 (12)	
O2	0.6657 (7)	0.1535 (4)	0.1739 (4)	0.0343 (12)	
O3	0.3924 (8)	0.2018 (4)	0.4107 (4)	0.0424 (13)	
O4	0.6947 (8)	0.2189 (4)	0.3891 (4)	0.0417 (13)	
O5	0.8814 (9)	0.1620 (5)	−0.0029 (5)	0.0533 (17)	
O6	0.5976 (8)	0.0707 (4)	−0.0775 (4)	0.0377 (12)	
O7	0.7609 (8)	−0.0319 (4)	0.0120 (4)	0.0425 (13)	
H7A	0.6855	−0.0566	0.0382	0.064*	
H7B	0.8141	−0.0784	−0.0152	0.064*	
O8	0.8336 (8)	0.0170 (5)	0.2355 (4)	0.0442 (14)	
O9	0.1307 (8)	0.0018 (5)	0.2258 (4)	0.0427 (14)	
O10	1.0501 (8)	0.2362 (5)	0.3173 (4)	0.0490 (15)	
H10A	1.1424	0.2256	0.3465	0.073*	
H10B	0.9746	0.2651	0.3505	0.073*	
C1	0.5762 (13)	0.2909 (6)	0.1437 (6)	0.0391 (18)	
C2	0.7517 (15)	0.3433 (7)	0.1545 (8)	0.053 (2)	
C3	0.790 (2)	0.4252 (8)	0.1307 (11)	0.086 (4)	
H3	0.9146	0.4587	0.1389	0.103*	
C4	0.635 (3)	0.4537 (10)	0.0945 (11)	0.097 (5)	
H4	0.6530	0.5102	0.0807	0.117*	
C5	0.455 (2)	0.4012 (10)	0.0783 (10)	0.082 (4)	
H5	0.3580	0.4227	0.0496	0.098*	
C6	0.396 (3)	0.3144 (9)	0.1011 (7)	0.105 (6)	
H6	0.2716	0.2803	0.0912	0.126*	
C7	0.5377 (11)	0.2061 (6)	0.1744 (5)	0.0306 (15)	
C8	0.5995 (12)	0.3522 (6)	0.5128 (6)	0.0380 (17)	
C9	0.4447 (16)	0.3912 (8)	0.5579 (7)	0.056 (2)	
H9	0.3217	0.3540	0.5411	0.067*	
C10	0.482 (2)	0.4869 (8)	0.6279 (8)	0.074 (4)	
H10	0.3817	0.5141	0.6570	0.089*	
C11	0.660 (2)	0.5406 (8)	0.6543 (9)	0.077 (4)	
H11	0.6803	0.6037	0.7021	0.093*	
C12	0.814 (2)	0.5043 (8)	0.6121 (8)	0.075 (4)	
H12	0.9369	0.5418	0.6296	0.090*	
C13	0.7761 (15)	0.4092 (8)	0.5425 (8)	0.058 (2)	
C14	0.5595 (11)	0.2510 (6)	0.4324 (5)	0.0328 (16)	
C15	−0.0168 (12)	−0.0643 (6)	0.3273 (6)	0.0349 (16)	
C16	0.1421 (14)	−0.1041 (7)	0.3439 (8)	0.054 (2)	
H16	0.2446	−0.0985	0.3111	0.064*	
C17	0.1563 (18)	−0.1518 (9)	0.4069 (9)	0.073 (3)	
H17	0.2674	−0.1756	0.4181	0.087*	
C18	0.0000 (17)	−0.1632 (8)	0.4532 (8)	0.065 (3)	
H18	0.0045	−0.1968	0.4945	0.078*	
C19	−0.1581 (15)	−0.1259 (8)	0.4385 (7)	0.057 (3)	
H19	−0.2618	−0.1333	0.4701	0.068*	
C20	−0.1660 (12)	−0.0773 (7)	0.3773 (6)	0.0426 (19)	
C21	−0.0202 (10)	−0.0121 (5)	0.2587 (5)	0.0292 (15)	
C22	0.7003 (14)	0.2145 (6)	−0.1082 (6)	0.046 (2)	
C23	0.849 (2)	0.2749 (9)	−0.1202 (9)	0.072 (3)	
C24	0.828 (3)	0.3368 (10)	−0.1663 (12)	0.108 (6)	
H24	0.9346	0.3763	−0.1729	0.129*	
C25	0.650 (3)	0.3397 (10)	−0.2026 (11)	0.113 (7)	
H25	0.6327	0.3816	−0.2348	0.136*	
C26	0.491 (2)	0.2812 (9)	−0.1926 (9)	0.088 (4)	
H26	0.3698	0.2864	−0.2180	0.105*	
C27	0.501 (3)	0.2134 (9)	−0.1455 (8)	0.101 (6)	
H27	0.3952	0.1736	−0.1392	0.121*	
C28	0.7316 (12)	0.1442 (6)	−0.0584 (6)	0.0389 (18)	

### Atomic displacement parameters (Å^2^)

**Table:**

	*U*^11^	*U*^22^	*U*^33^	*U*^12^	*U*^13^	*U*^23^
Pb1	0.02153 (14)	0.03441 (15)	0.03085 (15)	0.00615 (10)	0.00427 (10)	0.01469 (12)
Pb2	0.02253 (14)	0.05183 (19)	0.03065 (16)	0.00657 (12)	0.00338 (11)	0.01749 (14)
F1	0.098 (6)	0.095 (6)	0.116 (7)	−0.014 (5)	0.001 (5)	0.045 (5)
F2	0.087 (6)	0.111 (7)	0.172 (10)	−0.004 (5)	0.023 (7)	−0.014 (7)
F3	0.039 (3)	0.129 (6)	0.083 (4)	0.034 (3)	0.027 (3)	0.075 (4)
F4	0.120 (9)	0.226 (14)	0.235 (15)	−0.030 (9)	0.001 (10)	0.162 (13)
O1	0.025 (3)	0.051 (3)	0.044 (3)	0.008 (2)	0.009 (2)	0.023 (3)
O2	0.028 (3)	0.042 (3)	0.042 (3)	0.011 (2)	0.011 (2)	0.024 (3)
O3	0.031 (3)	0.049 (3)	0.038 (3)	0.002 (3)	0.006 (2)	0.008 (3)
O4	0.031 (3)	0.046 (3)	0.037 (3)	0.006 (2)	0.007 (2)	0.003 (3)
O5	0.042 (4)	0.074 (4)	0.047 (4)	−0.009 (3)	−0.009 (3)	0.037 (3)
O6	0.031 (3)	0.041 (3)	0.040 (3)	0.000 (2)	0.003 (2)	0.018 (3)
O7	0.036 (3)	0.041 (3)	0.046 (3)	0.006 (3)	0.008 (3)	0.013 (3)
O8	0.026 (3)	0.071 (4)	0.055 (4)	0.019 (3)	0.015 (3)	0.041 (3)
O9	0.028 (3)	0.055 (4)	0.055 (4)	0.010 (3)	0.011 (3)	0.031 (3)
O10	0.033 (3)	0.064 (4)	0.043 (3)	0.011 (3)	0.005 (3)	0.013 (3)
C1	0.053 (5)	0.034 (4)	0.036 (4)	0.019 (4)	0.018 (4)	0.014 (3)
C2	0.052 (6)	0.049 (5)	0.055 (6)	0.005 (4)	0.004 (5)	0.018 (5)
C3	0.111 (11)	0.047 (6)	0.111 (11)	0.003 (7)	0.049 (9)	0.039 (7)
C4	0.162 (16)	0.062 (8)	0.105 (11)	0.047 (10)	0.049 (11)	0.059 (8)
C5	0.125 (12)	0.069 (8)	0.077 (9)	0.052 (8)	0.024 (8)	0.044 (7)
C6	0.240 (19)	0.079 (8)	0.039 (6)	0.116 (11)	0.046 (8)	0.039 (6)
C7	0.031 (4)	0.038 (4)	0.022 (3)	0.006 (3)	0.004 (3)	0.010 (3)
C8	0.044 (5)	0.043 (4)	0.028 (4)	0.011 (4)	0.004 (3)	0.014 (3)
C9	0.063 (6)	0.059 (6)	0.045 (5)	0.016 (5)	0.016 (5)	0.014 (5)
C10	0.109 (10)	0.053 (6)	0.057 (7)	0.034 (7)	0.035 (7)	0.005 (5)
C11	0.126 (12)	0.041 (6)	0.053 (7)	0.011 (7)	0.020 (7)	0.004 (5)
C12	0.092 (9)	0.049 (6)	0.053 (7)	−0.018 (6)	0.006 (6)	−0.004 (5)
C13	0.054 (6)	0.063 (6)	0.055 (6)	0.014 (5)	0.016 (5)	0.018 (5)
C14	0.034 (4)	0.039 (4)	0.022 (3)	0.004 (3)	0.000 (3)	0.010 (3)
C15	0.039 (4)	0.038 (4)	0.030 (4)	0.010 (3)	0.003 (3)	0.015 (3)
C16	0.047 (5)	0.065 (6)	0.066 (6)	0.023 (5)	0.017 (5)	0.039 (5)
C17	0.074 (8)	0.090 (9)	0.083 (8)	0.038 (7)	0.009 (6)	0.059 (7)
C18	0.071 (7)	0.077 (7)	0.066 (7)	0.016 (6)	0.007 (6)	0.051 (6)
C19	0.054 (6)	0.074 (7)	0.054 (6)	0.004 (5)	0.007 (5)	0.039 (5)
C20	0.036 (4)	0.056 (5)	0.042 (5)	0.010 (4)	0.006 (4)	0.025 (4)
C21	0.025 (4)	0.031 (4)	0.034 (4)	0.005 (3)	0.006 (3)	0.015 (3)
C22	0.062 (6)	0.035 (4)	0.038 (5)	−0.005 (4)	0.006 (4)	0.015 (4)
C23	0.078 (9)	0.070 (7)	0.069 (8)	−0.010 (6)	−0.001 (6)	0.037 (6)
C24	0.141 (15)	0.075 (9)	0.111 (12)	−0.036 (9)	−0.004 (11)	0.065 (9)
C25	0.21 (2)	0.060 (8)	0.079 (10)	0.023 (11)	−0.002 (12)	0.043 (8)
C26	0.138 (13)	0.053 (7)	0.068 (8)	0.037 (8)	−0.017 (8)	0.019 (6)
C27	0.191 (15)	0.058 (7)	0.041 (6)	0.079 (9)	−0.037 (7)	0.000 (5)
C28	0.040 (4)	0.047 (5)	0.034 (4)	0.006 (4)	0.005 (3)	0.021 (4)

### Geometric parameters (Å, °)

**Table:**

Pb1—O4	2.480 (6)	C4—C5	1.36 (2)
Pb1—O2	2.489 (5)	C4—H4	0.9300
Pb1—O1	2.551 (6)	C5—C6	1.469 (18)
Pb1—O8	2.574 (5)	C5—H5	0.9300
Pb1—O9	2.621 (6)	C6—H6	0.9300
Pb1—O3	2.642 (6)	C8—C13	1.354 (13)
Pb1—O6^i^	2.766 (6)	C8—C9	1.420 (12)
Pb1—F3^ii^	2.856 (8)	C8—C14	1.513 (11)
Pb2—O2	2.517 (5)	C9—C10	1.394 (14)
Pb2—O7	2.534 (6)	C9—H9	0.9300
Pb2—O5	2.592 (6)	C10—C11	1.344 (18)
Pb2—O10	2.599 (6)	C10—H10	0.9300
Pb2—O8	2.603 (5)	C11—C12	1.385 (18)
Pb2—O9^ii^	2.670 (6)	C11—H11	0.9300
Pb2—O7^iii^	2.999 (6)	C12—C13	1.386 (14)
Pb2—O1^ii^	2.804 (5)	C12—H12	0.9300
F1—C2	1.261 (13)	C15—C16	1.381 (12)
F2—C13	1.294 (13)	C15—C20	1.389 (11)
F3—C20	1.351 (10)	C15—C21	1.502 (10)
F4—C23	1.290 (17)	C16—C17	1.381 (13)
O1—C7	1.239 (9)	C16—H16	0.9300
O2—C7	1.269 (9)	C17—C18	1.390 (16)
O3—C14	1.256 (9)	C17—H17	0.9300
O4—C14	1.256 (9)	C18—C19	1.347 (15)
O5—C28	1.227 (10)	C18—H18	0.9300
O6—C28	1.276 (10)	C19—C20	1.366 (12)
O7—H7A	0.8200	C19—H19	0.9300
O7—H7B	0.8200	C21—O8^iv^	1.243 (9)
O8—C21^ii^	1.243 (9)	C22—C23	1.366 (14)
O9—C21	1.247 (9)	C22—C27	1.467 (17)
O9—Pb2^iv^	2.670 (6)	C22—C28	1.517 (11)
O10—H10A	0.8200	C23—C24	1.353 (17)
O10—H10B	0.8200	C24—C25	1.35 (2)
C1—C2	1.344 (13)	C24—H24	0.9300
C1—C7	1.491 (10)	C25—C26	1.39 (2)
C1—C6	1.515 (17)	C25—H25	0.9300
C2—C3	1.386 (14)	C26—C27	1.433 (16)
C3—C4	1.37 (2)	C26—H26	0.9300
C3—H3	0.9300	C27—H27	0.9300
			
O4—Pb1—O2	74.52 (19)	C1—C2—C3	125.1 (11)
O4—Pb1—O1	81.3 (2)	C4—C3—C2	116.2 (13)
O2—Pb1—O1	51.13 (16)	C4—C3—H3	121.9
O4—Pb1—O8	83.7 (2)	C2—C3—H3	121.9
O2—Pb1—O8	68.63 (17)	C5—C4—C3	121.3 (11)
O1—Pb1—O8	119.76 (17)	C5—C4—H4	119.3
O4—Pb1—O9	130.77 (18)	C3—C4—H4	119.3
O2—Pb1—O9	120.83 (18)	C4—C5—C6	127.4 (13)
O1—Pb1—O9	77.90 (17)	C4—C5—H5	116.3
O8—Pb1—O9	145.0 (2)	C6—C5—H5	116.3
O4—Pb1—O3	50.68 (17)	C5—C6—C1	107.1 (14)
O2—Pb1—O3	106.06 (18)	C5—C6—H6	126.4
O1—Pb1—O3	72.71 (19)	C1—C6—H6	126.4
O8—Pb1—O3	131.93 (19)	O1—C7—O2	120.4 (7)
O9—Pb1—O3	80.51 (19)	O1—C7—C1	120.1 (7)
O6^i^—Pb1—F3^ii^	104.7 (2)	O2—C7—C1	119.6 (6)
O1—Pb1—O6^i^	85.90 (18)	C13—C8—C9	117.9 (9)
O2—Pb1—O6^i^	77.99 (18)	C13—C8—C14	123.1 (8)
O9—Pb1—O6^i^	69.51 (17)	C9—C8—C14	119.0 (8)
O8—Pb1—O6^i^	81.25 (18)	C10—C9—C8	118.2 (11)
O3—Pb1—O6^i^	146.29 (17)	C10—C9—H9	120.9
O4—Pb1—O6^i^	151.93 (18)	C8—C9—H9	120.9
O4—Pb1—F3^ii^	87.65 (20)	C11—C10—C9	121.4 (11)
O1—Pb1—F3^ii^	168.83 (21)	C11—C10—H10	119.3
O3—Pb1—F3^ii^	99.11 (19)	C9—C10—H10	119.3
O9—Pb1—F3^ii^	108.72 (18)	C10—C11—C12	121.7 (10)
O2—Pb1—F3^ii^	126.98 (22)	C10—C11—H11	119.2
O8—Pb1—F3^ii^	59.89 (19)	C12—C11—H11	119.2
O2—Pb2—O7	76.62 (18)	C11—C12—C13	116.6 (11)
O2—Pb2—O5	78.46 (19)	C11—C12—H12	121.7
O7—Pb2—O5	71.2 (2)	C13—C12—H12	121.7
O2—Pb2—O10	75.17 (18)	F2—C13—C8	123.0 (10)
O7—Pb2—O10	144.72 (19)	F2—C13—C12	112.9 (11)
O5—Pb2—O10	122.4 (2)	C8—C13—C12	124.1 (10)
O2—Pb2—O8	67.75 (17)	O4—C14—O3	122.0 (7)
O7—Pb2—O8	75.8 (2)	O4—C14—C8	118.9 (7)
O5—Pb2—O8	137.09 (18)	O3—C14—C8	119.1 (7)
O10—Pb2—O8	74.2 (2)	C16—C15—C20	115.2 (7)
O2—Pb2—O9^ii^	115.50 (16)	C16—C15—C21	120.1 (7)
O7—Pb2—O9^ii^	93.94 (19)	C20—C15—C21	124.7 (7)
O5—Pb2—O9^ii^	157.3 (2)	C17—C16—C15	123.2 (9)
O10—Pb2—O9^ii^	79.6 (2)	C17—C16—H16	118.4
O8—Pb2—O9^ii^	48.39 (16)	C15—C16—H16	118.4
O2—Pb2—O1^ii^	141.13 (16)	C16—C17—C18	118.2 (10)
O5—Pb2—O1^ii^	108.21 (19)	C16—C17—H17	120.9
O7—Pb2—O1^ii^	142.21 (17)	C18—C17—H17	120.9
O8—Pb2—O1^ii^	114.69 (18)	C19—C18—C17	120.3 (9)
O9—Pb2—O1^ii^	150.98 (13)	C19—C18—H18	119.8
O10—Pb2—O1^ii^	69.09 (17)	C17—C18—H18	119.8
O7^iii^—Pb2—O1^ii^	66.45 (15)	C18—C19—C20	120.0 (10)
O2—Pb2—O7^iii^	146.84 (16)	C18—C19—H19	120.0
O5—Pb2—O7^iii^	73.48 (19)	C20—C19—H19	120.0
O7—Pb2—O7^iii^	77.86 (17)	F3—C20—C19	116.8 (8)
O8—Pb2—O7^iii^	125.04 (17)	F3—C20—C15	120.2 (7)
O9—Pb2—O7^iii^	138.80 (13)	C19—C20—C15	123.0 (8)
O10—Pb2—O7^iii^	135.54 (17)	O8^iv^—C21—O9	120.6 (7)
C7—O1—Pb1	92.7 (5)	O8^iv^—C21—C15	121.9 (6)
C7—O2—Pb1	94.9 (4)	O9—C21—C15	117.5 (7)
C7—O2—Pb2	150.1 (5)	C23—C22—C27	121.0 (11)
Pb1—O2—Pb2	114.7 (2)	C23—C22—C28	122.1 (10)
C14—O3—Pb1	89.7 (4)	C27—C22—C28	116.9 (9)
C14—O4—Pb1	97.4 (5)	F4—C23—C24	114.8 (13)
C28—O5—Pb2	135.0 (5)	F4—C23—C22	121.2 (11)
Pb2—O7—H7A	109.5	C24—C23—C22	124.0 (14)
Pb2—O7—H7B	112.5	C25—C24—C23	118.3 (14)
H7A—O7—H7B	101.3	C25—C24—H24	120.8
C21—O8—Pb1^ii^	171.78 (14)	C23—C24—H24	120.8
C21—O8—Pb2^ii^	169.90 (14)	C24—C25—C26	121.1 (12)
Pb1—O8—Pb2	109.0 (2)	C24—C25—H25	119.4
C21—O9—Pb1	146.2 (5)	C26—C25—H25	119.4
C21^iv^—O9—Pb2	161.84 (13)	C25—C26—C27	123.8 (15)
Pb1^iv^—O9—Pb2	152.03 (16)	C25—C26—H26	118.1
Pb2—O10—H10A	109.5	C27—C26—H26	118.1
Pb2—O10—H10B	129.8	C26—C27—C22	111.7 (15)
H10A—O10—H10B	115.8	C26—C27—H27	124.1
C2—C1—C7	123.9 (8)	C22—C27—H27	124.1
C2—C1—C6	122.7 (10)	O5—C28—O6	125.0 (8)
C7—C1—C6	113.3 (9)	O5—C28—C22	118.3 (8)
F1—C2—C1	121.4 (9)	O6—C28—C22	116.7 (7)
F1—C2—C3	113.4 (11)		

Symmetry codes: (i) −*x*+1, −*y*, −*z*; (ii) *x*+1, *y*, *z*; (iii) −*x*+2, −*y*, −*z*; (iv) *x*−1, *y*, *z*.

### Hydrogen-bond geometry (Å, °)

**Table:**

*D*—H···*A*	*D*—H	H···*A*	*D*···*A*	*D*—H···*A*
O7—H7A···O6^i^	0.82	2.15	2.881 (8)	148
O7—H7B···O5^iii^	0.82	2.66	3.360 (8)	144
O10—H10A···O3^ii^	0.82	2.07	2.892 (8)	174
O10—H10B···O4	0.82	2.22	2.856 (8)	135
O10—H10B···F2	0.82	2.44	3.161 (13)	147
C19—H19···O3^v^	0.93	2.56	3.364 (13)	145

Symmetry codes: (i) −*x*+1, −*y*, −*z*; (iii) −*x*+2, −*y*, −*z*; (ii) *x*+1, *y*, *z*;  (v) −*x*, −*y*, −*z*+1.

## References

[bb1] Higashi, T. (1995). *ABSCOR* Rigaku Corporation, Tokyo, Japan.

[bb2] Morsali, A. & Mahjoub, A. R. (2005). *Solid State Sci.* **7**, 1429–1437.

[bb3] Rigaku (1998). *PROCESS-AUTO* Rigaku Corporation, Tokyo, Japan.

[bb4] Sheldrick, G. M. (2008). *Acta Cryst.* A**64**, 112–122. 10.1107/S0108767307043930 18156677

[bb5] Xiao, H.-P. & Morsali, A. (2007). *Solid State Sci.* **9**, 155–158.

[bb6] Zhang, B.-S. (2004). *Z. Kristallogr. New Cryst. Struct.* **219**, 483–484.

[bb7] Zhang, B.-S. (2005). *Z. Kristallogr. New Cryst. Struct.* **220**, 73–74.

[bb8] Zhang, B.-S. (2006*a*). *Acta Cryst.* E**62**, m2645–m2647.

[bb9] Zhang, B.-S. (2006*b*). *Z. Kristallogr. New Cryst. Struct.* **221**, 191–194.

[bb10] Zhang, B.-S. (2006*c*). *Z. Kristallogr. New Cryst. Struct.* **221**, 355–356.

[bb11] Zhang, B.-S., Zhu, X.-C., Yu, Y.-Y., Chen, L., Chen, Z.-B. & Hu, Y.-M. (2005). *Z. Kristallogr. New Cryst. Struct.* **220**, 211–212.

[bb12] Zhu, H.-G., Xu, Y., Yu, Z., Wu, Q.-J., Fun, H.-K. & You, X.-Z. (1999). *Polyhedron*, **18**, 3491–3495.

